# Gloving in medicine: a boon for infection prevention or a hindrance?

**DOI:** 10.1017/ash.2025.10145

**Published:** 2025-10-29

**Authors:** Diana Vilar-Compte, Pamela Garciadiego-Fossas, Cyntia Ibanes-Gutiérrez

**Affiliations:** 1 Hospital Epidemiology and Infection Control, Department of Infectious Diseases, Instituto Nacional de Cancerologia, Mexico City, Mexico; 2 Hospital Epidemiology and Infection Control, Department of Infectious Diseases, Instituto Nacional de Enfermedades Respiratorias, Mexico City, Mexico; 3 Hospital Epidemiology and Infection Control, Department of Infectious Diseases, Instituto Nacional de Pediatria, Mexico City, Mexico

## Introduction

Practicing modern medicine without gloves as personal protective equipment (PPE) is unimaginable. The introduction of gloves into medical practice in the 19th century significantly reduced infections in healthcare settings.

The World Health Organization (WHO)^1^ and other medical societies endorse using gloves to lower the risk of contamination by blood and other body fluids and decrease the transmission of microbes between patients and healthcare workers.

Over time, the widespread use of gloves has increased due to a better understanding of how infections spread and a heightened focus on patient safety during medical care. Additionally, the emergence of the HIV epidemic in the late 20th century, along with the associated risk of bloodborne transmission, led to a significant boost in glove usage. The H1N1 influenza pandemic and Ebola also increased glove use, dramatically escalating during the COVID-19 pandemic. However, this excessive usage has also raised awareness of the environmental impact of disposable gloves.

When the WHO published its hand hygiene guidelines in 2009,^1^ it outlined the indications for using gloves in various medical scenarios. However, over time—especially following the influenza and COVID-19 pandemics—gloves have frequently been misused and overused, leading to increased cross-infections, unnecessary costs, and excessive disposal. These issues have significant ecological consequences and pose new challenges for healthcare facilities and the medical gloves market.

Based on the available evidence and recommendations, glove usage in 21st-century medical practice must be reassessed. This reassessment should ensure **safe patient care** while promoting **rational and environmentally sustainable programs** to mitigate glove-related pollution and other personal protective equipment waste.

## History of glove use in medicine

The history of glove use in medicine highlights the evolution of medical practices, prevention, and infection control. It unfolds across a spectrum of historical contexts, all rooted in the 19^th^ century’s burgeoning interest in hygiene and antisepsis.

The first rudimentary medical gloves were made in 1758 from the cecum of a sheep. The gloves were used by a German physician, Johan Walbaum, for gynecological examination and deliveries.^2^ Still, it was not until the mid-1850s that Dr. William Halsted from Johns Hopkins introduced them to surgery to protect his chief surgical nurse hands, Caroline Hampton (who later became his wife), from a combination of chemicals.

Nurse Hampton was known for her dexterity and efficiency, but her hands suffered badly, enduring rashes, eczema, and pain. Halsted and Hampton developed a novel solution for her: appropriate protective gloves.^3^ By that time, Charles Goodyear had revolutionized the rubber industry with the discovery of the vulcanization process for rubber in 1844, leading to the creation of medical gloves. The Goodyear Rubber Company commissioned the first pair of gloves. In 1890, the surgical gloves we recognize today were introduced into the operating room by Halsted and Hampton. These gloves covered Caroline’s hands and forearms, finally protecting her from the harmful chemicals in the operating room.^2^

In 1893, Joseph Bloodgood, a former disciple of Halsted and the first surgeon to wear gloves regularly—not only to protect the surgeon’s hands but also to prevent infections in patients—published the first study on surgical rubber gloves. He wore gloves during hernia operations and observed a significant decline in postsurgical infections. He demonstrated that the rate of surgical infections after hernia procedures using gloves was 2%, compared to over 17% in surgeries without gloves.^2,4^ Additionally, at the end of the 19^th^ century, Jan Mikulicz-Radecki from Poland began performing surgeries with sterilized cotton gloves, further contributing to the revolution of medical gloves. Later, he introduced rubber gloves into surgery and cover shoes in the operating theater.^5^

The use of gloves in surgery was slow, as many surgeons feared that it would cause a loss of touch and blunt that crucial guide to operating back in those days. Some detractors argued that any gains from the lower infection rates would not outweigh the risk of blind touch surgery, and not every surgeon adopted this practice.^3^

## New materials and the use of gloves in the medical profession beyond surgery

With the rise of synthetic materials, glove innovations emerged during the 20^th^ century. Neoprene gloves were introduced during World War II but had little impact on medical practice. The true and most significant change in glove materials occurred in the 1960s when latex rubber gloves and gamma radiation were introduced, a technology still utilized for the sterilization of disposable gloves. Latex gloves became a major milestone, enhancing the safety and comfort of medical procedures and becoming the standard in most hospitals.^6^

During the 1960s and 1970s, guidelines and protocols on glove use were developed, reflecting a global understanding of the importance of hygiene and prevention in daily medical practice.^7^

With the increasing exposure to latex gloves, latex allergy rose among healthcare workers. It was not until the 1990s that less allergenic synthetic materials, such as nitrile and polyurethane, were introduced into glove technology. Nitrile gloves are increasingly popular, as they are less allergenic and provide the same protection as latex gloves, with almost half of the market nowadays.^8^

## Gloves as a protection barrier

With the emergence of *Staphylococcus aureus* as a hospital pathogen in the 1960s, hospitals in the US and Europe developed the first infection control programs. In 1968, the first edition of the American Hospital Association´s manual presented a barrier precautions scheme for patients with communicable diseases, listing the need for gloves, gowns, masks, and visitor screening.

Around 1985, the acquired immunodeficiency syndrome epidemic impacted isolation procedures due to the risk associated with blood and blood-containing fluids. This resulted in the formulation of Standard Precautions, which emphasize the importance of hand hygiene and the use of PPE such as gloves for routine medical care.^7,9^

Over the past few decades, isolation guidelines have evolved significantly, mainly because of the recognized risk of infection transmission within the healthcare setting. Also, the H1N1 influenza pandemic in 2009 and the COVID-19 pandemic significantly increased the use of gloves as part of PPE. At the same time, their inappropriate use in different settings and the environmental impact they cause have also raised concerns.

**Figure 1.**
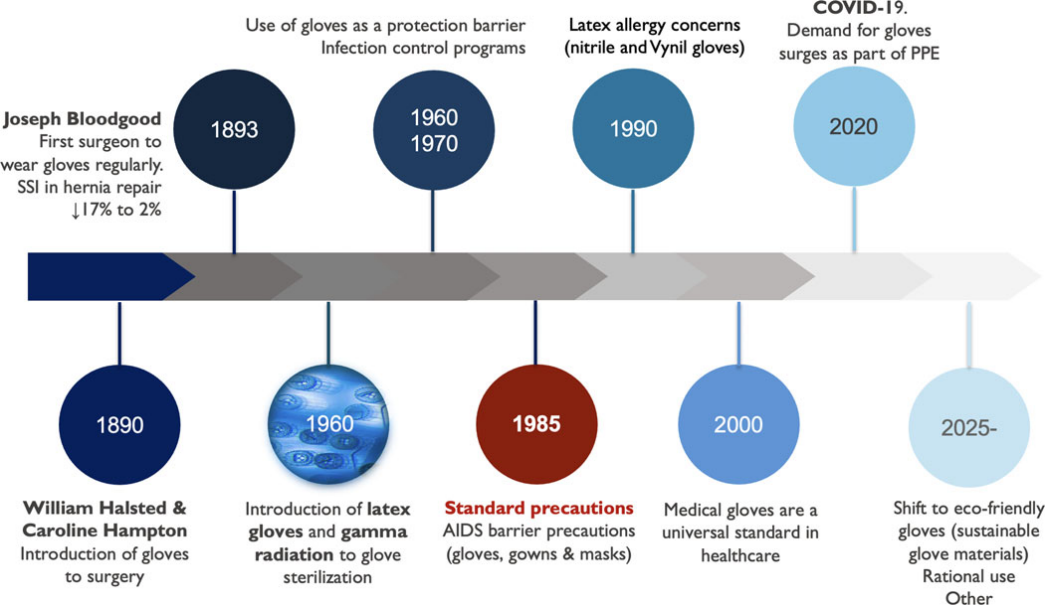
Milestones in the history of gloves in medicine.

With the evolution of PPE and a better understanding of pathogen transmission, gloves are undoubtedly important as protective barriers. Still, their use and misuse in the first quarter of the 21^st^ century must be revised for us as medical professionals and the planet’s health. In Figure 1, milestones on the use of gloves from the 19^th^ century to 2025 are depicted.

## Policies and recommendations for glove use in medicine

**Figure 2.**
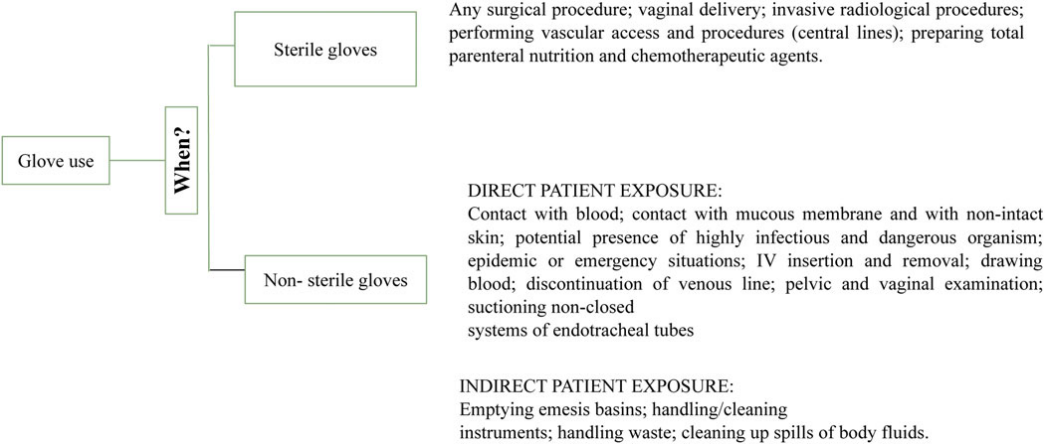
Recommendations for glove use.

Hand hygiene is the most effective strategy for preventing healthcare-related infections. This measure is often closely linked to gloves, so healthcare workers must fully understand the two indications for using gloves in clinical settings^1,10^

a. To reduce the risk of contamination of the healthcare workers’ (HCWs) hands with blood or other body fluids, including contact with mucous membranes and non-intact skin.

b. When recommended as part of contact precautions, to minimize the risk of transmitting infectious agents to the patient’s environment and other patients. In Figure 2, we illustrate the proper use of gloves as outlined by the WHO.

The choice between wearing sterile or non-sterile gloves is determined by the nature of the task undertaken by HCWs. Sterile gloves are indicated for most surgical and invasive procedures as part of aseptic technique and sterile procedures. They should be worn for vaginal deliveries, spinal, epidural, and caudal procedures, the preparation of chemotherapeutic agents, and the preparation of total parenteral nutrition.^11^

Non-sterile gloves are utilized to minimize the risks during activities that might lead to hand contamination from blood or other bodily fluids, as well as contact with mucous membranes and non-intact skin. They are also a key recommendation for contact precaution protocols, preventing the spread of microorganisms in the environment and to other patients^1,12^

### Contact precautions and the use of gloves

Gloves and gowns are used as contact precautions to prevent the transmission of multidrug-resistant organisms (MDROs) and other concerning microorganisms such as *Clostridioides difficile* and resistant *Candida* species. They are also recommended for patients with uncontrolled secretions or fluids.^13^ However, significant controversy surrounds the relative advantages and disadvantages of contact precautions.^14,15^

Several cluster-randomized trials examining methicillin-resistant *S. aureus* (MRSA) acquisition, which included contact precautions, did not find a significant reduction in either MRSA infection or acquisition.^16-18^ Additionally, a time series study^19^ and a systematic review didn’t show significant difference in SARM or vancomycin-resistant Enterococci (VRE) rates following the discontinuation of contact precautions.^20^

The most recent recommendations by SHEA/IDSA/APIC and the European Centre for Disease Prevention and Control for preventing MRSA transmission in acute care hospitals align with the continued use of gloves and gowns for patients colonized or infected with MRSA.^21^ However, some countries, such as Switzerland^12^ and France^22^ no longer recommend the routine use of gloves during contact precautions for MRSA.

With increased access to alcohol-based hand rubs and various studies on the effectiveness of gloves and gowns, hospitals that implement robust horizontal precautions no longer experience ongoing MRSA outbreaks and maintain stable or low rates of MRSA infections; they no longer routinely require contact precautions for MRSA control.^15^ Contact precautions should be discontinued for patients with MRSA (and most likely with VRE) and only be used as an additional approach, not as an essential practice. For other microorganisms, such as MDR gram-negative organisms, resistant *Candida* species, or emerging pathogens, the use of gowns and gloves remains a cornerstone for preventing their transmission.

## Inappropriate uses for gloves

**Figure 3.**
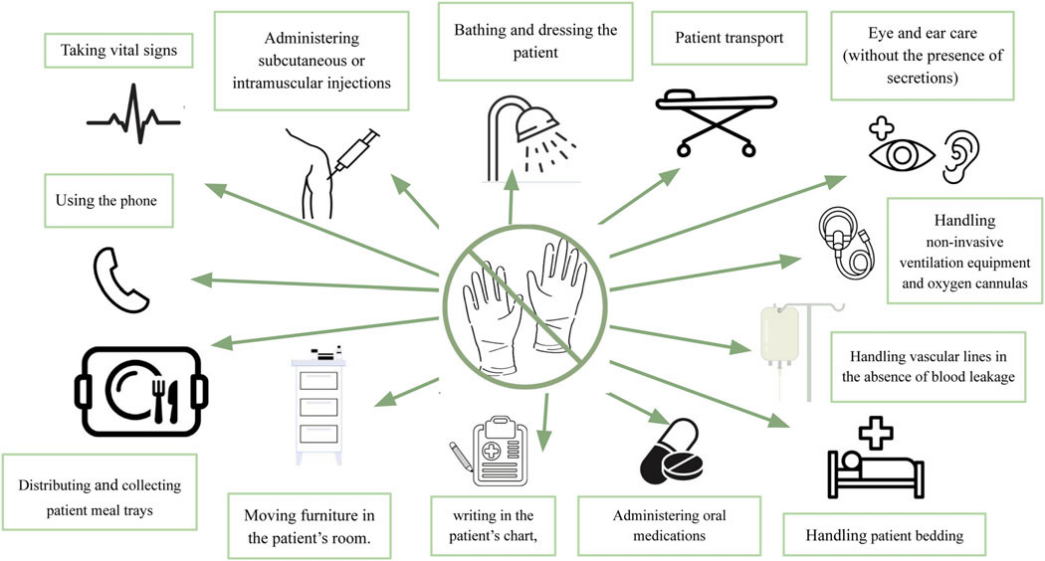
Situations where gloves are not routinely recommended, **except** for patients in contact precautions.

The decision to wear gloves is often influenced by emotions and a misjudgment of personal risk rather than a desire to protect patients.^23^ Although the 2009 WHO hand hygiene guidelines specify the tasks for which gloves are not indicated^1^ it is common for HCWs and others, such as fast-food handlers,^24^ to wear gloves for tasks where they are unnecessary, sometimes for extended periods. This practice can lead to decreased handwashing and an increased risk of hand contamination and transmission. Situations where gloves are unnecessary according to guidelines are shown in Figure 3. Avoiding the excessive and improper use of gloves is just as important as their proper use.

Although gloves can reduce hand contamination during patient care,^25^ they can also create a false sense of security that encourages inappropriate use and, paradoxically,^12^ decrease hand hygiene compliance. Multiple studies indicate that improper glove use during patient care heightens the potential for cross-contamination and the risk of healthcare-associated infections. Reported glove contamination rates range from 8 to 69%, as shown in Table 1.^26,27^

**Table 1.** Rates of glove contamination after contact with microorganisms

Reference	year	Type of microorganism	Source		Frequency of glove contamination after interaction
Boyce JM,et al.^55^	1997	MRSA	Contaminated surfaces.		42%
Ray,A. et al.^56^	2002	VRE	Contaminated environmental surfaces in the rooms of colonized patients.		46%
Grabsch E, et al.^57^	2006	VRE	Patients currently or previously colonized with VRE who undergo outpatient procedures (consultations, radiology procedures, and hemodialysis).		8–16%
Hayden. et al.^58^	2008	VRE	VRE colonized patient and their environment.	55 and 29	66–69%
Snyder, G, et al.^35^	2008	MRSA and VRE	Patients with MRSA and/or VRE and their environment.	137	MRSA: 18% VRE 8%
Morgan et al.^34^	2010	MDR *Acinetobacter baumannii* and MDR *Pseudomonas aeruginosa*	Patients colonized with MDR *A. baumannii* or colonized with both MDR *A. baumannii* and MDR *P. aeruginosa.*		MDR *A baumannii*: gloves: 36%. Hands after glove removal: 4.5% MDR *P aeruginosa*: Gloves: 6.7%. Hands after glove removal: 1%
Morgan, et al.^59^	2012	MRSA, VRE, MDR *Pseudomonas aeruginosa*, MDR *Acinetobacter baumannii*	ICU Patients with MDR bacteria within in the last year.		11.2%, 10%, 17.4% and 29.3% respectively Hands after glove removal: 3.3%, 1.7%, 3.5%, 4.2%, respectively
Guerrero et al.^60^	2012	*Clostridioides difficile*	Skin of patients with *Clostridioides difficile* and environmental surfaces in their rooms.		50% in both (skin and environmental surface)
Rock et al.^61^	2014	*Klebsiella KPC* *Klebsiella non-KPC*	Patients with *Klebsiella* and their environment.		*Klebsiella* KPC: 10.4% *Klebsiella* non-KPC: 16.9%
Roghmann MC, et al.^62^	2015	MRSA	Residents and HCW from 13 community-based nursing homes.		24%

Adapted from:.^26^ MRSA: methicillin-resistant *Staphylococcus aureus; VRE:* Vancomycin Resistant Enterococci; MDR: multi-drug resistant; PA: Pseudomonas aeruginosa; AB: *Acinetobacter baumannii.* No study mentions adherence to hand hygiene.

Picheansathian and Chotibang, in their systematic review, report an average of 61.2% adherence to glove utilization among HCWs. The compliance with glove use among doctors was significantly lower than that of nurses and healthcare assistants. The rate of glove use was highest in the intensive care unit (65.2%) and lowest in pediatric wards (9.1%). Additionally, gloves were used in 16.7% of contacts without a clinical indication for using gloves. Conversely, gloves were not used in 21.1% of high-risk contacts when their use would have been indicated.^28^

Loveday’s study revealed that gloves are not only misused, but also that in 37% of glove-use incidents, the risk of cross-contamination increased, most often due to failure to change gloves or perform hand hygiene after glove doffing.^29^ Girou demonstrated that the failure to change or remove contaminated gloves was the most significant factor in poor hand hygiene, resulting in a high risk of microorganism transmission. He reported that 1 in 5 contacts between staff and patients involved possible transmission of microorganisms. After contact, 59% of gloves were contaminated with the same bacteria that were colonizing or infecting the patient.^30^

Hand hygiene is recommended before donning and after doffing gloves; however, it has been reported that only 18% of HCWs clean their hands prior to using gloves, and only 27% adhere to proper glove-changing protocols.^31^ This lack of hand hygiene before putting on gloves can lead to the introduction of commensal and pathogenic bacteria into glove boxes, indicating that unused, non-sterile gloves are potential vehicles for pathogen transmission in hospitals.^32^ Furthermore, the longer gloves are worn, the greater the risk of micro-perforations and, consequently, of pathogen transmission. One of the most common causes of glove misuse is their prolonged use without replacement during tasks not intended for glove usage (e.g., distributing or collecting patient dietary trays).^12,23^ Lindberg et al. report continued glove use in approximately half of the observed episodes of patient care, with an average of 3.3 surfaces being touched by gloves that should have been removed or changed.^33^

Several studies suggest that gloves may contribute to increased transmission or outbreaks of MDROs. Morgan et al.^34^ observed in a prospective cohort study that the gloves and gowns of HCWs were often contaminated with MDR *A. baumannii,* and to a lesser extent with MDR *P. aeruginosa,* during routine care of patients infected or colonized with these organisms. The hands of HCWs were found to be contaminated with MDR *A. baumannii* after nearly 5% of contacts following glove removal. Additionally, Snyder et al. found that 17%–18% of healthcare workers attending patients with MRSA and/or VRE acquired the microorganisms via gloves or gowns.^35^ Regarding carbapenem-resistant Enterobacteriaceae, a study that cultured gloves and gowns of healthcare personnel after contact with 313 colonized patients found glove contamination in 7.9% of cases.

## Hand hygiene before gloving

Hand hygiene is always necessary before donning sterile gloves for invasive procedures. For nonsterile gloves used in routine care, recent evidence indicates that hand hygiene immediately before gloving may not significantly decrease contamination and can sometimes be skipped—especially if gloves are used properly.^36,37^ Since most indications for glove use happen right before patient contact, hand hygiene still remains essential in most cases. Based on current knowledge, the only possible exception is when donning gloves for contact precautions before touching only the patient’s environment—not the patient directly—provided that hand hygiene is performed after removing the gloves.

## ABHR application over gloves during prolonged use

During glove use, in specific situations where changing or removing gloves for hand hygiene is impractical—such as during task-dense periods in the operating room or as a contingency during outbreaks or glove shortages—targeted use of alcohol-based hand rub (ABHR) on gloved hands may be considered to reduce pathogen transmission.

Current clinical guidelines do not universally recommend routine use of ABHR on gloves during patient care. The standard recommendations are that hand hygiene with ABHR should be performed before donning gloves and after removing gloves.

However, recent updates and select studies have addressed the targeted use of ABHR on gloves in specific clinical scenarios. The 2022 SHEA/IDSA/APIC practice recommendations discuss disinfecting gloves with ABHR during task-intensive clinical care (e.g., anesthesia induction or extubation), during outbreaks, or when there are shortages of personal protective equipment. The practice is referenced in the context of extended glove use and doffing procedures for high-consequence pathogens, with the caveat that prolonged glove use and repeated disinfection may increase the risk of glove perforation.^10,38^

## Reuse and disinfection of gloves

Although medical gloves are intended for single use, the concept of decontamination and reuse is not new and has emerged during exceptional circumstances, particularly during extreme shortages such as those experienced during the COVID-19 pandemic or in managing high-risk pathogens like Ebola, where glove removal may lead to hand contamination. Despite being common in low-resource settings with supply limitations, the WHO explicitly states that glove reprocessing is not recommended due to the absence of standardized, validated, and affordable procedures. WHO emphasizes preventive strategies such as staff education on appropriate glove use, timely stock replenishment, and the procurement of high-quality disposable gloves to eliminate the need for reuse.^39^ Similarly, during the pandemic, the U.S. Centers for Disease Control and Prevention temporarily issued guidance allowing glove decontamination in certain circumstances; however, this guidance has since been withdrawn, and to date, no medical glove has been approved for reuse.

Esmizadeh et al suggested that reusing gloves might contribute to reducing medical waste; however, the chemicals and energy required for proper decontamination could surpass the environmental benefit of glove disposal while potentially increasing the risk of pathogen transmission.^40^ Experimental studies, such as that performed by Dawson et al., have evaluated the physical integrity of medical gloves following various decontamination methods. In their study, they assessed how different disinfection treatments—specifically soap and water, alcohol-based hand sanitizer, chlorine, and quaternary ammonium compounds—affected glove durability. The outcomes focused on glove tearing or breakage showed that performance after decontamination varied significantly depending on the glove material, method used, and number of decontamination cycles. Vinyl gloves demonstrated a high rate of failure, with frequent tearing across all methods, while latex surgical gloves maintained their integrity more effectively. The authors emphasized that, although their findings provide insight into physical barrier compromise, further research is needed to evaluate microbiological safety, tensile strength, and elasticity to determine the viability of glove reuse in clinical settings.^41^ The 2022 SHEA/IDSA/APIC Practice Recommendations on hand hygiene clearly specify that these are approaches that should not be considered a routine part of hand hygiene.^10^

## The environmental impact of gloving in the healthcare industry

The widespread use of gloves carries significant ecological consequences, including the production of non-biodegradable waste and greenhouse gas emissions throughout their lifecycle.^42,43^ The COVID-19 pandemic exacerbated these challenges, with unprecedented increases in glove usage worsening the environmental burden.^44^ For example, glove usage in the English health services more than tripled during the pandemic, rising from 1,763,164,000 used gloves in 2019 to 5,492,770,000 from February 2020 to February 2021.^45^ The following sections explore strategies for reducing glove consumption and waste without compromising infection prevention and control standard.

## Educational interventions and behavioral changes

One of the most effective ways to reduce glove use is through rational, evidence-based guidelines,^46^ that can help optimize usage while ensuring safety. For instance, combining proper glove use with stringent hand hygiene practices can ensure adequate infection prevention while minimizing waste.^1^

The successful implementation of these guidelines relies on the education and engagement of HCWs, highlighting the dual importance of safety and environmental stewardship. Training programs should include modules on the proper use of gloves, the significance of hand hygiene as a safety measure, and the environmental implications of disposable gloves. Furthermore, implementation science offers a framework to identify barriers, such as the fear of compromising patient safety or insufficient institutional support, as well as facilitators like strong leadership commitment, clear protocols, and peer accountability, to design interventions that are context-specific and effective. By employing implementation science methodologies, healthcare organizations can systematically assess the feasibility, acceptability, and sustainability of initiatives aimed at reducing glove overuse, while ensuring that clinical safety remains uncompromised through outcome measurement.^47^

The environmental impact of gloves is driven by their reliance on materials such as nitrile, latex, and vinyl, which are non-biodegradable.^48^ The development of biodegradable gloves made from plant-based polymers or modified natural rubber offers a promising alternative through reducing long-term pollution.^49^ However, biodegradable gloves must adhere to strict safety, durability, and functionality standards to ensure they can effectively replace traditional gloves without compromising clinical care. Additionally, the production of these gloves should emphasize sustainable manufacturing processes.

Effective waste management systems are critical for mitigating the environmental impact of glove disposal. Proper segregation of medical waste, including gloves, from general waste is essential to prevent contamination and facilitate recycling or energy recovery.^50^ Recycling initiatives for materials such as nitrile and latex have shown potential in reducing glove waste volume, though these programs are not yet widely implemented due to logistical and cost barriers.^51^ Additionally, advanced incineration technologies with emission controls can convert glove waste into energy while minimizing the release of harmful byproducts. To become scalable solutions, these systems require investment and collaboration among healthcare facilities, policymakers, and waste management industries. Moreover, attention must be given to the waste generated by the packaging of sterile gloves, which contributes to a higher waste.

## Enhancing hand hygiene practices. is AI an opportunity?

Promoting hand hygiene according to WHO recommendations as the cornerstone of infection prevention is another strategy to reduce reliance on gloves.^39^ By strengthening hand hygiene practices and restricting glove use to high-risk scenarios, healthcare facilities can lower glove consumption while maintaining infection control standards.

Integrating artificial intelligence (AI) into hand hygiene monitoring systems offers significant potential to improve adherence and technique among HCWs. By utilizing AI-equipped cameras, compliance with hand hygiene protocols can be continuously monitored, allowing accurate data collection to assess and enhance adherence.^52^ One notable example was evaluated by Lacey, et.al. in 2020, which provides real-time feedback on hand hygiene techniques. This automated system helps users identify errors and improve their technique immediately, ensuring a higher standard of hand hygiene.^53^ Furthermore, the incorporation of AI technologies in these devices not only monitors compliance but also offers objective feedback, ultimately contributing to optimizing clinical practices and reducing the risk of healthcare-associated infections.

## System-level compromise

Achieving sustainable glove use requires a comprehensive approach that integrates policy changes, technological innovation, and collaboration across the healthcare sector. Policymakers can encourage the adoption of biodegradable materials and sustainable manufacturing practices through subsidies or regulatory frameworks.^54^ At the institutional level, hospitals can form sustainability committees to develop and monitor initiatives aimed at reducing the environmental footprint of disposable gloves and other medical supplies. Collaboration with manufacturers, waste management providers, and environmental organizations can drive the development and implementation of scalable solutions.

In summary, gloves are an essential protective measure for patients and HCWs and help decrease infections, but we must follow evidence and use them only as recommended. We should reconsider their use in contact precautions, especially for methicillin-resistant *S. aureus*. The environmental impact of disposable gloves in healthcare is a urgent concern that needs a thoughtful and balanced approach. By promoting proper glove use, investing in biodegradable materials, improving waste management, and strengthening hand hygiene practices, healthcare systems can significantly reduce their ecological footprint. Education and system-level strategies, including AI, are essential for integrating sustainability into healthcare culture without sacrificing safety. Through these combined efforts, the healthcare sector can continue to protect patients and HCWs while helping balance global environmental sustainability with patient care priorities.
